# Assessing the repeatability of absolute CMRO_2_, OEF and haemodynamic measurements from calibrated fMRI

**DOI:** 10.1016/j.neuroimage.2018.02.020

**Published:** 2018-06

**Authors:** Alberto Merola, Michael A. Germuska, Kevin Murphy, Richard G. Wise

**Affiliations:** a Cardiff University Brain Research Imaging Centre (CUBRIC), School of Psychology, Cardiff University, UK; b Max Planck Institute for Human Cognitive and Brain Sciences, Leipzig, DE, Germany; c Cardiff University Brain Research Imaging Centre (CUBRIC), School of Physics and Astronomy, Cardiff University, UK

## Abstract

As energy metabolism in the brain is largely oxidative, the measurement of cerebral metabolic rate of oxygen consumption (CMRO_2_) is a desirable biomarker for quantifying brain activity and tissue viability. Currently, PET techniques based on oxygen isotopes are the gold standard for obtaining whole brain CMRO_2_ maps. Among MRI techniques that have been developed as an alternative are dual calibrated fMRI (dcFMRI) methods, which exploit simultaneous measurements of BOLD and ASL signals during a hypercapnic-hyperoxic experiment to modulate brain blood flow and oxygenation.

In this study we quantified the repeatability of a dcFMRI approach developed in our lab, evaluating its limits and informing its application in studies aimed at characterising the metabolic state of human brain tissue over time. Our analysis focussed on the estimates of oxygen extraction fraction (OEF), cerebral blood flow (CBF), CBF-related cerebrovascular reactivity (CVR) and CMRO_2_ based on a forward model that describes analytically the acquired dual echo GRE signal.

Indices of within- and between-session repeatability are calculated from two different datasets both at a bulk grey matter and at a voxel-wise resolution and finally compared with similar indices obtained from previous MRI and PET measurements. Within- and between-session values of intra-subject coefficient of variation (CV_intra_) calculated from bulk grey matter estimates 6.7 ± 6.6% (mean ± std.) and 10.5 ± 9.7% for OEF, 6.9 ± 6% and 5.5 ± 4.7% for CBF, 12 ± 9.7% and 12.3 ± 10% for CMRO_2_. Coefficient of variation (CV) and intraclass correlation coefficient (ICC) maps showed the spatial distribution of the repeatability metrics, informing on the feasibility limits of the method.

In conclusion, results show an overall consistency of the estimated physiological parameters with literature reports and a satisfactory level of repeatability considering the higher spatial sensitivity compared to other MRI methods, with varied performance depending on the specific parameter under analysis, on the spatial resolution considered and on the study design.

## Highlights

• Two datasets (N1 = 10 and N2 = 16) from dual calibrated fMRI experiments with respiratory tasks are considered.
• Maps of OEF, CBF, CVR and CMRO2 calculated with a recently proposed estimation approach are shown.
• Estimated indices of global and voxel-wise repeatability are reported for within- and between- session designs.
• Results show a good trade-off between repeatability and spatial resolution when compared to previous studies.

## Introduction

Brain activity is reliant on energy release principally through oxidative metabolism. For this reason, a number of MRI methods are under development to directly quantify the rate of cerebral metabolic oxygen consumption (CMRO_2_). CMRO_2_ offers a marker of the physiological state of brain tissue (Lin et al., 2010), with potential applications in tumour (Brown and Wilson, 2004), stroke (Derdeyn et al., 2002), neurological (Santens et al., 1997) and neurodegenerative disorders (Ishii et al., 1996).

PET imaging based on an oxygen isotope (^15^O) is often still regarded as the gold standard for obtaining whole brain CMRO_2_ maps despite the technical complexity, the risks related to the administration of ionising radiation and the implicit limits for longitudinal studies. Recent MRI methods for measurement of CMRO_2_ have been introduced based on exploiting the magnetic field differences between the superior sagittal sinus (Jain et al., 2010) or major veins (Fan et al., 2012) and the surrounding parenchyma, T2-oxygenation calibration curves refined with velocity selective techniques (Bolar and Rosen, 2011, Guo and Wong, 2012) or quantifying venous oxygen saturation via the T2 of venous blood (Lu and Ge, 2008, Xu et al., 2009). While this last approach is limited to bulk level estimates, it is currently found to show the highest level of precision and repeatability (Liu et al., 2013).

Another group of techniques, known as calibrated BOLD methods, aims to estimate CMRO_2_ from BOLD and arterial spin labelling (ASL) signals, exploiting respiratory tasks and mathematical models describing the complex relationship between oxygen metabolism, BOLD signal and cerebral blood flow (CBF) in the brain. Recently, extensions of the original approaches of Davis and Hoge (Davis et al., 1998, Hoge et al., 1999) have been developed allowing the use of both hypercapnia and hyperoxia induced CBF and BOLD signal changes within the same experiment, to estimate cerebral venous deoxyhaemoglobin concentration and thus oxygen extraction fraction (OEF) and absolute CMRO_2_ (Bulte et al., 2012, Gauthier and Hoge, 2012, Wise et al., 2013), an approach also known as quantitative O_2_ imaging (QUO2) or dual calibrated fMRI (dcFMRI).

Eliminating the use of PET ionising agents in mapping CMRO2 is desirable, although one of the factors that currently limit the application of dcFMRI methods in clinical research studies is the lack of characterisation of their variability and repeatability. In fact, to our knowledge only a single study based on a dcFMRI technique (Lajoie et al., 2016) has been recently presented reporting repeatability measurements. This involved a cohort of eight healthy subjects undergoing two separate dcFMRI scan sessions (within 24 h) and the data were analysed with a QUO2 estimation approach (Gauthier and Hoge, 2012), supplying estimates of whole brain grey matter and regional repeatability.

Our study also focuses on characterising the repeatability of the dcFMRI technique, although considering estimates obtained from dcFMRI experiments with a novel estimate approach based on a forward model recently developed in our lab (Germuska et al., 2016). This model allows us to describe analytically the contributions of BOLD signal, ASL signal and of the measured end-tidal partial pressures of CO_2_ and O_2_ to the measured dual echo GRE signal in a dcFMRI acquisition, at a voxel-wise level of resolution (see Appendix for more details). We are therefore able to present quantitative maps of four main physiological parameters involved in brain metabolism across grey matter: OEF, CBF, CO_2_-induced perfusion cerebrovascular reactivity (CVR) and CMRO_2_.

Our aim is to evaluate the reliability of the estimates and to collect reference data to evaluate the limits and the viability of the estimation framework for adoption in future studies aimed at characterising the metabolic state of human brain tissue. Compared to the work of Lajoie and colleagues (Lajoie et al., 2016), a more extended cohort of subjects and set of measurements are considered for this study. Indices quantifying within-session repeatability of the estimates are presented, based on measurements from a test-retest experiment on ten healthy volunteers in the resting state. A second group of indices quantifying between-session repeatability of the results is also presented, based on a previously published study on sixteen healthy volunteers exploiting the same dcFMRI analysis framework but a crossover design with repeated measurements (Merola et al., 2017). We quantify repeatability both at a whole grey matter level and at a voxel-wise level, supplying a good level of spatial detail for such measurements.

## Materials and methods

### Participants and experimental design

Exclusion criteria were introduced with special attention to possible difficulties in complying with respiratory tasks (asthma, smoking, cold/flu, etc.) and known cardio/cerebrovascular disease. Volunteers' tolerance of hypercapnic periods and prolonged breathing through a facial mask was tested with a benching session held in the days before the scanning session. The study was approved by the local ethics committee and written informed consent was obtained from each participant.

**Fig. 1:**
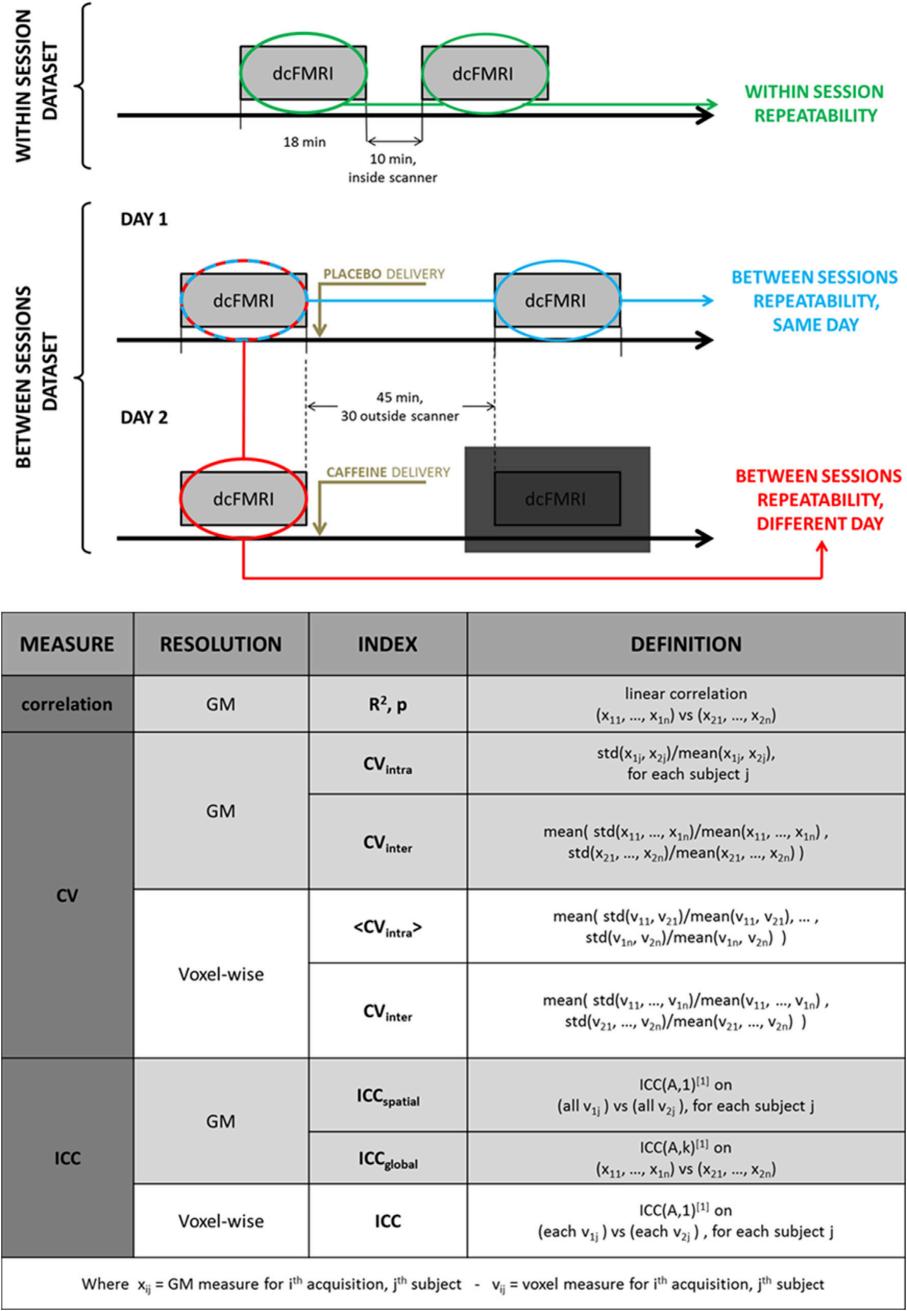
Top: diagram showing the experimental design for the within-session and between-sessions datasets. Bottom: list of indices calculated for each measure, both at whole grey matter (GM) and voxel-wise resolution. All indices were calculated for every repeatability considered: within session, between sessions - same day and between sessions – different day. [1] (McGraw and Wong, 1996).

For **within-session** assessments ten healthy volunteers (4 females, age = 27.4 ± 10) were recruited. Each participant was scanned at rest (eyes open) in a single scan session (see Fig. 1, top). A dual calibrated fMRI scan (dcFMRI scan, 18 min) was performed and then repeated after about 10 min. During each of the dcFMRI scans an 18 min respiratory task was delivered, with interleaved levels of hypercapnia, hyperoxia and medical air being delivered to the subjects. We will refer to this as the within-session repeatability dataset.

For **between-session** assessments a second set of measurements is included from a previously presented dcFMRI study on the acute effects of caffeine for which sixteen healthy participants (8 females, age = 24.7 ± 5.1) were recruited (Merola et al., 2017). The results from one subject were excluded, due to the degraded nature of the data (please refer to the original paper (Merola et al., 2017)). In this case each participant was scanned on two different days (30.1 ± 18.8 days apart, same time of the day), each day including the same protocol with a first scan session followed by the delivery of a capsule of drug or placebo outside the scanner and then a second scan session 45 min later (see schematic in Fig. 1). Crucially in each day the dcFMRI acquisitions were run in two separate scanning sessions, with the participant spending time outside the MR suite in between them. For the purpose of this study only the pre-dose and placebo sessions were considered (see schematic in Fig. 1) to avoid the caffeine effect. Each session included a dcFMRI acquisition with specifications and respiratory tasks identical to the ones used for the within-session repeatability dataset. We will refer to this as the between-session repeatability dataset.

### Gas delivery, breathing circuit and respiratory task

**Fig. 2:**
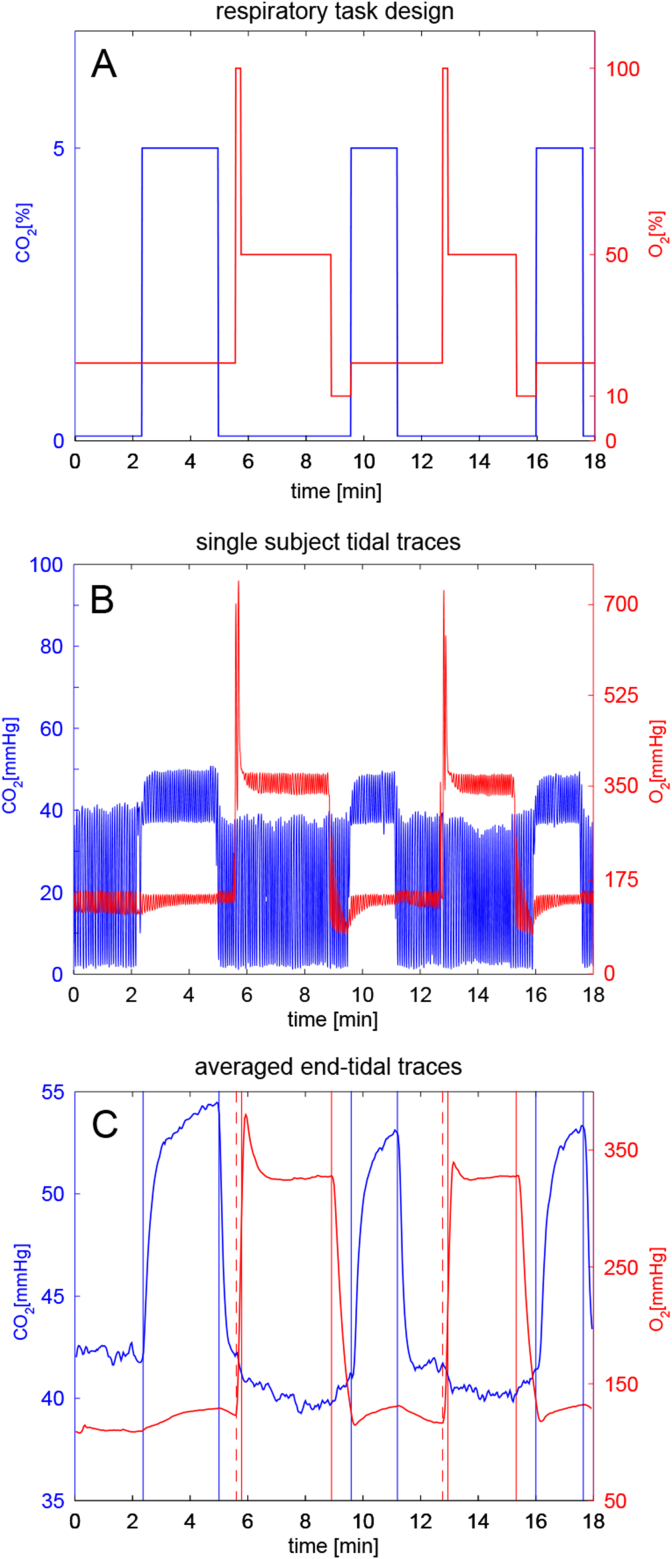
A - Inspired gas fractions during the respiratory task. B – Tidal traces of a single representative subject. C - End-tidal traces averaged across all subjects and sessions of the within-session dataset. Vertical lines highlight the timing of the respiratory task. In both B and C periods of hypercapnia and of hyperoxia are clearly visible, interleaved with short periods of normocapnia-normoxia. Positive and negative emphases can be distinguished before and after the plateau hyperoxic periods, respectively. As expected, periods of hyperoxia appear to produce a reduction in end-tidal CO_2_ and periods of hypercapnia are associated with slight increases in end-tidal O_2_.

The respiratory task design we adopt is similar to interleaved paradigms previously presented in literature (Bulte et al., 2012, Wise et al., 2013) and was optimized using noise modelling as previously described (Germuska et al., 2016). The design includes three periods of hypercapnia interleaved with two periods of hyperoxia, for a total duration of 18 min (see Fig. 2, A). In order to achieve hypercapnia, fixed values of 5% CO_2_ (balance air) were administered. Inspired fractions of 50% O_2_ (balance air) were delivered to achieve hyperoxia. Although in this last case, the levels of administered gas were modified with positive and negative emphasis; short periods of respectively 100% O_2_ (14s) and 10% O_2_ (40s) were delivered in order to accelerate the process of reaching the hyperoxic state and the return to normoxia (see Fig. 2). Although hypoxic mixtures were administered, their short duration did not induce arterial hypoxia, as monitored by a pulse oximeter attached to the volunteers' finger. Mixtures of 5% CO_2_ (balance air), 10% O_2_ (balance N_2_), 100% O_2_ and medical air were delivered at a total flow rate of 25 l/min to the gas mixing chamber which was placed in the MR control room. The mixing chamber was then connected to the breathing circuit through a humidifier. An independent O_2_ backup cylinder was also connected directly to the breathing circuit. The gas delivery system consisted of a laptop personal computer using in-house Matlab software (Mathworks, Natick, MA, USA) to control the voltage output from a NI-DAQ AD converter (National Instruments, Austin, TX). The output voltages were then fed into four mass flow controllers (MKS Instruments, Wilmington, MA, USA) that allowed us to administer the desired gas mixture. The respiratory circuit adopted was similar to the one proposed by Tancredi and colleagues (Tancredi et al., 2014). This circuit includes a system of one-way valves that minimizes re-breathing and an open reservoir that allows the subject to breathe room air when flow ceases to the circuit. Air was sampled from the volunteers' tight-fitting facemask and tidal partial pressures of O_2_ and CO_2_ were measured and recorded using rapidly responding gas analysers (AEI Technologies, Pittsburgh, PA, USA).

### Data acquisition

For both datasets presented, scanning was performed on a 3T GE HDx MRI system (GE Healthcare, Milwaukee WI) with a body transmit coil and 8-channel head receive coil. All participants underwent (or had available) whole brain T1-weighted structural scans (3D FSPGR, 1 × 1 × 1 mm voxels, TI/TR/TE = 450/7.8/3 ms).

dcFMRI acquisitions were acquired for both within- and between-session repeatability datasets collecting simultaneous perfusion and BOLD imaging data with a PASL PICORE, QUIPSS II imaging sequence with a dual-gradient echo (GRE) readout and spiral k-space acquisition with the following parameters: TE_1_ = 2.7 ms, TE_2_ = 29 ms, TR = 2.2 s, Flip Angle = 90°, FOV = 22 cm, Matrix = 64 × 64, 12 slices of 7 mm thickness with an inter-slice gap of 1 mm acquired in ascending order, TI_1_ = 700 ms, TI_2_ = 1500 ms for the most proximal slice and was incremented for the subsequent slices, tag thickness = 20 cm, 10 mm gap between labelling slab and bottom slice, 10 cm QUIPSS II saturation band thickness. This resulted in a 490-volume acquisition (245 tag-control pairs) for each of the dcFMRI acquisitions.

All dcFMRI scans were preceded by two calibration scans. The first consisted of a single shot scan to estimate the equilibrium magnetization of brain tissue (M_0_), used for perfusion quantification (Çavuşoǧlu et al., 2009), with the same acquisition parameters as for the perfusion-weighted scans, except for being acquired with fully relaxed magnetization and no labelling. The second was a low resolution, minimal contrast image used for coil sensitivity correction (Wu et al., 2011), with the same acquisition parameters as for the equilibrium magnetization scan, except for TE = 11 ms and TR = 2 s.

### Data analysis

#### dcFMRI data and end-tidal traces

dcFMRI data were pre-processed with motion correction (MCFLIRT (Jenkinson et al., 2002),) and brain extraction (BET (Smith, 2002),) and spatially smoothed with a Gaussian kernel of 6 mm with SUSAN (Smith and Brady, 1997), separately for echo 1 and echo 2. Estimation of physiological parameters of interest was performed with the forward model previously developed in our lab (Germuska et al., 2016) adapted for a Bayesian approach. This model - described in the Appendix section - was adopted because it allows us to take into account different aspects of physiology contributing to the measured BOLD and ASL signals in a simultaneous optimization and also because it is less prone to estimation failure compared to previous calibrated fMRI methods (Germuska et al., 2016). The priors on estimates were defined specifying means and standard deviations (mean, std.) as OEF = (0.35, 0.1), CBF = (60, Inf) ml/100 g/min, CVR = (3, 0.774) %mmHg, where by “Inf” we mean a non-informative prior. These values were fixed in agreement with reported physiological ranges and consistently with those used in the original study on the Bayesian framework for the forward model (Germuska et al., 2016). A prior was also defined on the estimated parameter K = (0.07,0.087), as for a recent study from our centre (Merola et al., 2017). Non-informative priors are used to initialize the estimate without carrying information, therefore they can be thought of as uniform distributions of probability. No prior is defined on the estimates of CMRO_2_ as this is calculated as CMRO_2_ = CBF·OEF·CaO_2_ (see Eq. A-11 in Appendix), where CaO_2_ is the arterial content of oxygen. With regards to the remaining parameters, they were kept the same as those adopted in the original work (Germuska et al., 2016) as also reported in Table 2 in the Appendix.

The inputs to the framework are dual echo GRE images and, PetO_2_ and PetCO_2_ traces. Analytic models (see Equations 1–8 in (Germuska et al., 2016) and the Appendix section) describing the magnetization decay occurring at the first and second TE were used to estimate grey matter maps of OEF, CBF, CVR and CMRO_2_. As for the parameters α and β used in Eq. 5 of (Germuska et al., 2016), they were fixed to 0.06 and 1 respectively, following the results of our previous optimisation study (Merola et al., 2016). Prior to analysis, the end-tidal responses were visually aligned with the MR data to remove the influence of any bulk delay between the recorded end-tidal traces and the fMRI data. Possible alignment errors amount to fractions of TR and likely have a negligible effect on the final estimation. Low-resolution functional images (mean TE_1_ across time) were co-registered to the high-resolution T1 weighted anatomical space using FSL FLIRT (Jenkinson et al., 2002) with 6 DOF for each subject. Registrations from the individual anatomical space to the MNI space were calculated with FNIRT (Andersson et al., 2007) for second level analysis. The parametric maps obtained from the analysis pipeline in the low-resolution functional space, were finally expressed into the MNI space using the calculated spatial transformations.

Mean grey matter values of each estimated parameter were calculated for the scans from an inclusive joint mask defined by I) partial volume grey matter values (based on the individual FSL FAST segmented high resolution anatomical maps) greater than 50%, II) estimated values of CBF within the range [0200] ml/100 g/min. The first criterion was imposed as an empirical threshold to avoid values affected by poor SNR of the signal in white matter, while the second was used to exclude non-physiological values, likely associated with high noise in some areas of the ASL images. A small proportion of voxels for which the estimation algorithm did not converge were also excluded from the analysis.

#### Within-session repeatability analysis

Indices quantifying the repeatability of the estimates were calculated for each physiological parameter both at a bulk grey matter and the voxel-wise level and are summarised in the table of Fig. 1.

Firstly indices were calculated at a bulk grey matter level with a correlation analysis between the estimates at the two time points: coefficient of determination (R^2^) and statistical significance (p).

Then the intraclass correlation coefficient, or ICC (McGraw and Wong, 1996, Shrout and Fleiss, 1979), was used as a measure of absolute agreement between the bulk estimates. The ICC has previously been applied to fMRI data to quantify the ratio between the data variance of interest and the total data variance (Bright and Murphy, 2013, Lipp et al., 2015). In particular, it can be applied in a voxel-wise fashion in order to obtain estimates of spatial repeatability of the signal (Lipp et al., 2014). Two different ICC indices were therefore considered: one calculated on whole grey matter values of the parameters across subjects (corresponding to ICC(A,k) in (McGraw and Wong, 1996)) and another considering voxel-wise comparisons between the two scans for each participant separately (corresponding to ICC(A, 1) in (McGraw and Wong, 1996)). These are hence referred to as “global ICC” (ICC_global_) and “spatial ICC” (ICC_spatial_) respectively (see table in Fig. 1). Both are interpreted according to commonly used guidelines that classify values of ICC below 0.4 as “poor”, values between 0.41 and 0.59 as “fair”, values between 0.60 and 0.74 as “good” and values > 0.74 as “excellent” (Cicchetti, 2001).

In order to evaluate the spread of the bulk estimates around their mean values, coefficients of variation (CV) of the estimates were also calculated. Two CV indices were considered: one taking account of the differences between the subjects of the cohort (i.e. inter-subjects, CV_inter_) and the other considering the variability occurring in each subject separately (ie intra-subject, CV_intra_, see table in Fig. 1). CV_inter_ was calculated as the mean of the CV values calculated for the two sessions, each of which was obtained as the ratio between the standard deviation and mean. CV_intra_ was calculated for each person by dividing the standard deviation of the estimates from two sessions by their mean. These CV indices were also visually represented in Bland-Altman plots, scatterplots in which the differences between two set of measurements are plotted against their means. Calculating the mean (m) and standard deviation (std) across the differences, it is then possible to characterise as outliers the values lying beyond the interval of m ± 1.96 × std.

The relationship between the similarity of the respiratory traces in the two acquisitions, expressed as a correlation, and the estimated indices of CV_intra_ was investigated, looking for possible sources of nuisance influencing intra-session variability.

Indices were also calculated at a voxel-wise level. Maps of the ICC index for each estimated parameter were calculated measuring the absolute agreement between each voxel across subjects (corresponding to ICC(A, 1) in (McGraw and Wong, 1996)). As for the bulk case, two indices were considered for CV: CV_inter_ was calculated as the mean between the CV (=std/m) values calculated for each of the two sessions. CV_intra_ maps were also calculated for each subject as the ratio between the standard deviation and the mean of the two measurements. Differently from the bulk estimates, a single map denoted <CV_intra_> was then calculated as the mean of each subject's CV_intra_ map, for an easier comparison with the CV_inter_ map (see table in Fig. 1).

#### Between-session repeatability analysis

Between-sessions repeatability was assessed similarly to the within sessions analysis but considering the dataset from our previous study on caffeine effects (see schematic in Fig. 1).

In order to avoid the effects of caffeine, only the three acquisitions without caffeine administration were considered in this case (i.e. both acquisitions in day 1 and the first in day 2, see Fig. 1). Therefore in this instance two different sets of measurements were calculated: the first comparing data acquired in different sessions but on the same day, which we shall refer to as “same day, between sessions repeatability” (or “between, same day”), while the second comparing different sessions and different days, which we shall refer to as “different day, between sessions repeatability” (or “between, different day”, see Fig. 1).

At a bulk level, measurements of between-session correlation were calculated: CV (CV_intra_ and CV_inter_) and ICC (ICC_global_ and ICC_spatial_). Then maps of the CV (<CV_intra_> and CV_inter_) and ICC indices were also calculated. All measurements are defined as for the within session case and are given for both same day- and different day-, between sessions repeatability.

The code used in this manuscript for data analysis is openly available from the Cardiff University data archive http://doi.org/10.17035/d.2017.0041693648. However, due to ethical considerations open access cannot be given to the in vivo subject data or data derived from this.

## Results

### Within-session dataset

#### Respiratory traces and bulk results

The mean baseline PetO_2_ value was 113 mmHg, while it was 42 mmHg for PetCO_2_. Plateau levels of hyperoxia caused a mean increase of approximately 230 mmHg in PetO_2_ and the mean increase in PetCO_2_ from baseline with hypercapnia was 11.5 mmHg. Moreover, periods of hyperoxia appear to produce a reduction in PetCO_2_ of about 2 mmHg, while periods of hypercapnia showed an increase in PetO_2_ of approximately 10 mmHg, consistently with literature findings (Floyd et al., 2003; Tancredi et al., 2014). An example of tidal measurements from a single subject and the averaged end-tidal traces for this dataset.

With regards to grey matter values of the four estimated physiological parameters, means calculated across subjects show only slight and not significant differences between the two time points, with pooled mean values of 0.38 (SEM ± 0.024) for OEF, 56 (SEM ± 3.8) [ml/100 mg/min] for CBF, 2.6 (SEM ± 0.15) [%/mmHg] for CVR and 183 (SEM ± 16) [μmol/100 mg/min] for CMRO_2_.

**Fig. 3:**
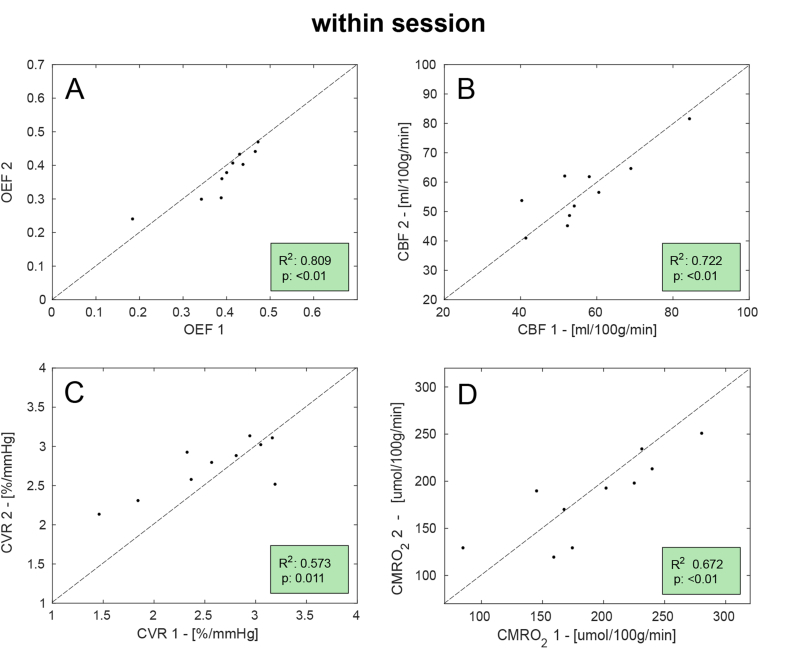
Scatterplots for the correlation analysis between the two sets of measurement (denoted as 1 and 2) of the within-session dataset. Dotted lines show unity and also displayed are the coefficient of determination (R^2^) and statistical significance (p).

Results of the correlation analysis are reported for all parameters in Fig. 3. In this case the goodness of fit is mixed: while OEF and CBF show relatively high values of the coefficient of determination (R^2^ > 0.7), CVR and CMRO_2_ only show moderate agreement between the two measurements (R^2^ > 0.5).

**Fig. 4:**
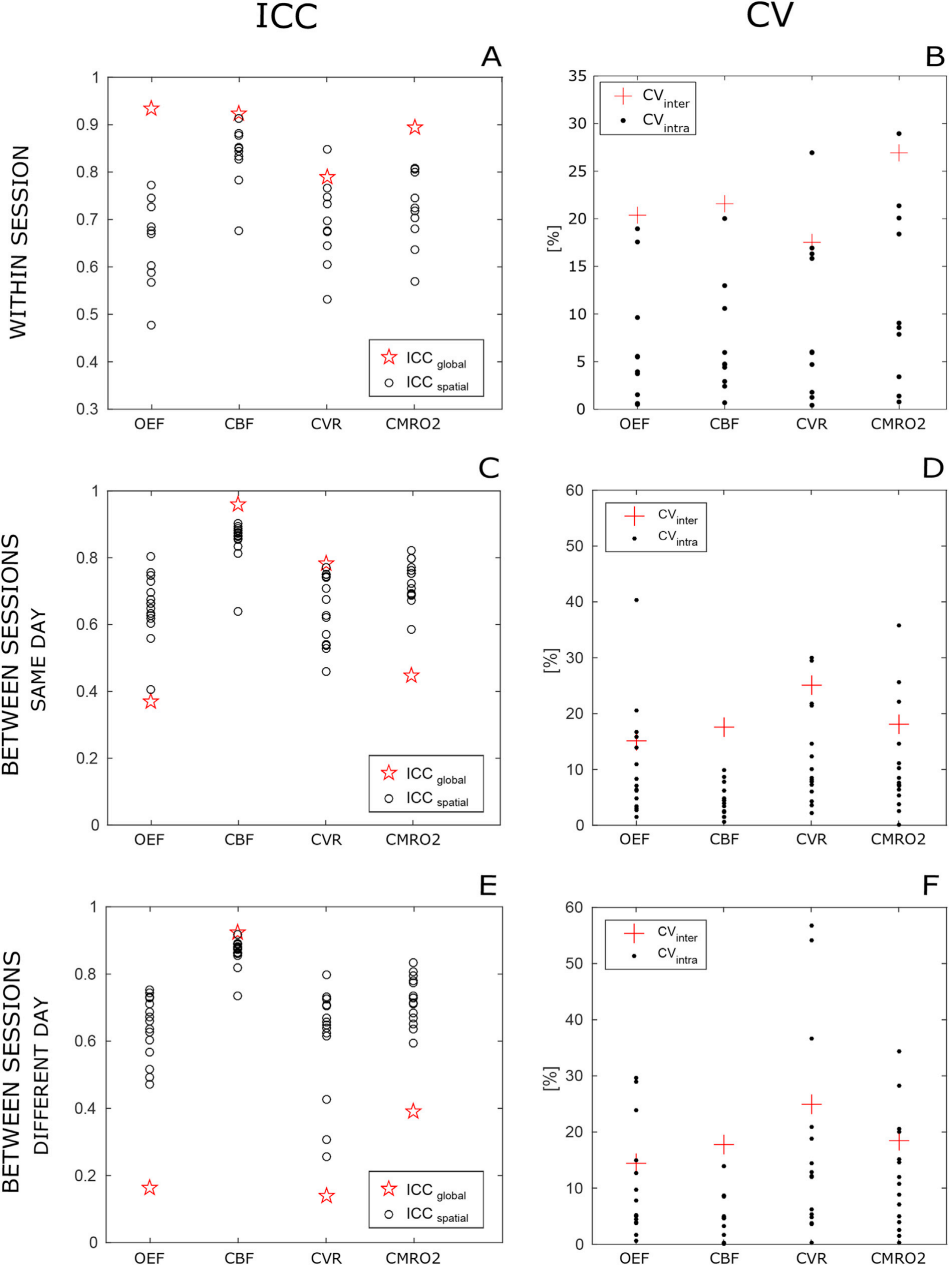
ICC and CV indices calculated at a grey matter level for all estimated parameters. A,B: within session dataset; C,D: between sessions, same day dataset; E,F: between sessions, different day dataset. Indices for individual subjects (CV_intra_ and ICC_global_) are shown in black circles and dots while group indices (CV_inter_ and ICC_global_) are shown in red stars and crosses respectively for ICC and CV. CV_intra_ is the intra-subject CV and CV_inter_ is the inter-subjects CV; ICC_global_ is the ICC(A,k) calculated between subjects at a GM level and ICC_spatial_ is the ICC(A, 1) calculated within subjects across voxels.

With regards to the ICC indices, ICC_global_ is found to be “excellent” for all four parameters, with particularly high performances for OEF and CBF (>0.9, Fig. 4A). Results are more varied for the voxel-wise analysis, with values of ICC_spatial_ remarkably high for CBF, mostly “excellent” for CVR and CMRO_2_, while mostly “good” for OEF.

Fig. 4B also shows CV indices. Values of CV_inter_ are generally high, ranging between 17.5% for CVR to 26.9% for CMRO_2_. CV_intra_ indices have similar distributions across parameters, with a value (mean ± std.) of 6.7 ± 6.6% for OEF, 6.9 ± 6% for CBF, 9.5 ± 8.8% for CVR and 12 ± 9.7% for CMRO_2_. In only three cases (not corresponding to the same subject) CV_intra_ is higher than CV_inter_.

Figure S1 in the Supplementary Material reports the linear relationship between the similarity of the respiratory traces in the two sessions - calculated as correlation - and the estimated indices of CV_intra_ for OEF. Results show a significant negative association among the two, although this effect is not found for the remaining physiological parameters estimated. Notably the two subjects with highest CV_intra_ also show the lowest values of correlation for CO_2_ and O_2_ traces between runs.

Bulk results visualised with Bland-Altman plots (reported in the Supplementary Material section, Figure S2) show most of the values clustering around the pool means for OEF and CVR, with bias in the differences of 4.9% and −6.5% respectively compared to the relative mean. For these parameters it is also possible to find an outlier (not always corresponding to the same subject). Distributions for CBF and CMRO_2_ are instead broader, with bias in the differences of just −0.4% and 4.6% respectively and no apparent outliers.

#### Voxel-wise results

**Fig. 5:**
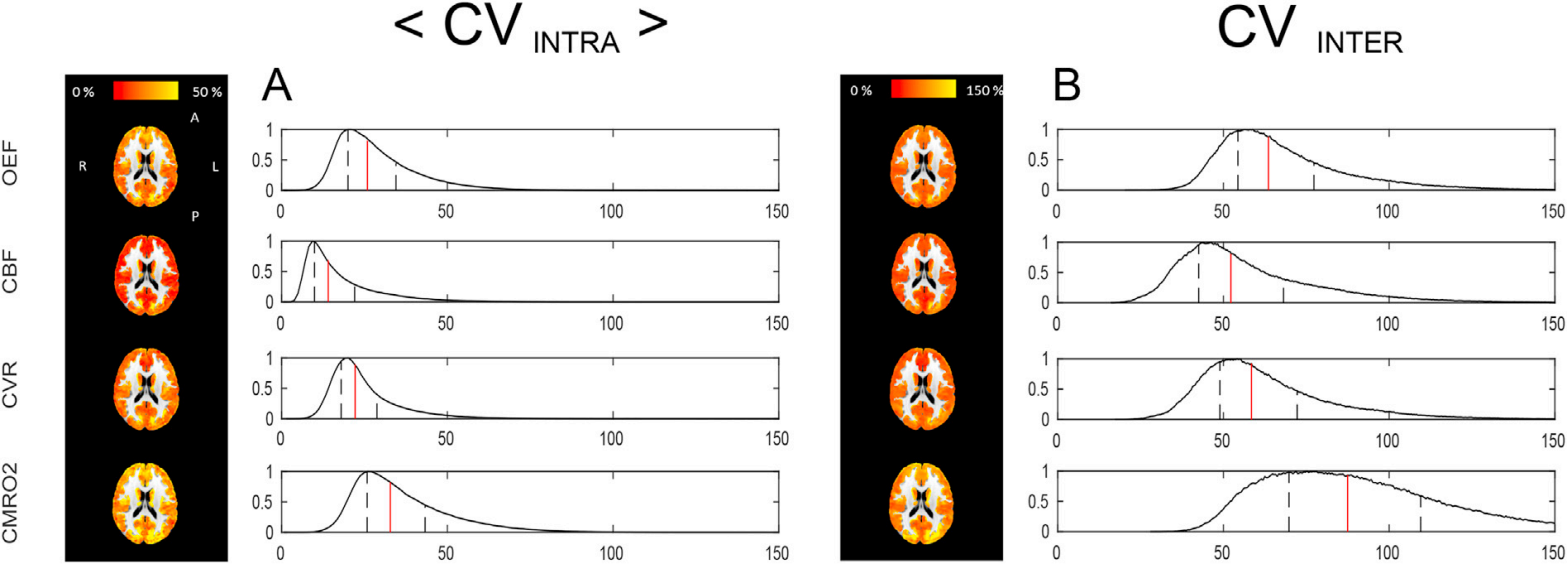
Voxel-wise CV indices calculated from the within-session dataset. A: results for <CV_intra_>, the mean across subjects of the intra-subject CV. B: results for CV_inter_, the inter-subject CV. For both, reported are the axial views of the calculated maps for each physiological parameter and relative histograms showing the distributions of the calculated values (in red the median and in black the interquartile range limits).

Maps of the CV indices at a voxel-wise level and their normalised histograms are reported in Fig. 5. As for the bulk estimates, values of the mean of the intra-subject CV are generally lower than values of inter-subjects CV. Notably, for all physiological parameters areas of interface between grey matter and different structures (white matter, ventricles and skull) present higher CV values. For both <CV_intra_> and CV_inter_, CBF shows the lowest variability, with values mostly homogeneous across parameters apart from few focal areas. A similar situation is shown by OEF and CVR, but with higher estimates. CMRO_2_ shows instead high <CV_intra_> and CV_inter_ indices with irregular distributions in space. The histograms support such evidence, with positively skewed distributions and median values lying around 25% for <CV_intra_> while above 50% for CV_inter_.

Finally, Figure S3 in the Supplementary Material section shows maps of the ICC index for each parameter and normalised histograms. The spatial distribution of the ICC is similar to that of the CV indices, with lower values associated with areas of interface between grey matter and different structures and CBF results showing the higher degree of uniformity. This is supported by the distributions, which all present negative skewness and with the best performance associated with the estimates of CBF reporting a median of 0.76 (classifiable as “excellent”), while for the remaining parameters the median lies between 0.55 for CVR and 0.60 for CMRO_2_.

### Between-sessions dataset

#### Bulk results

The effects elicited by the respiratory task are consistent with those found for the within-session repeatability dataset, with mean baseline values were 113 mmHg for PetO_2_ and 39 mmHg for PetCO_2_, with mean changes from baseline due to hypercapnia and hyperoxia of 12 mmHg and 211 mmHg respectively.

**Fig. 6:**
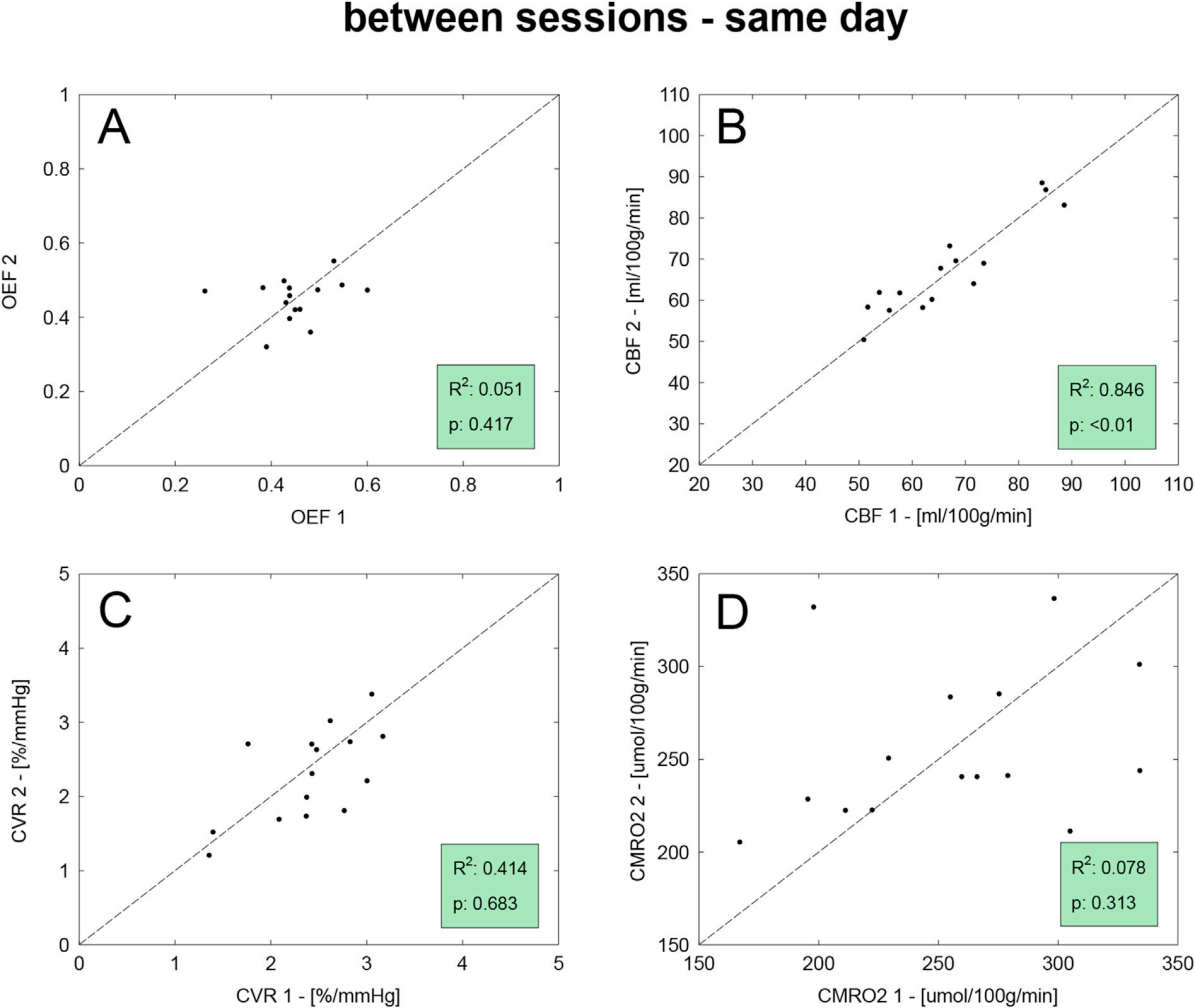
Results of the repeatability analysis on the between-sessions dataset with measurements acquired in the same day. Scatterplots for the correlation analysis between the two sets of measurement (denoted as 1 and 2), for all four estimted physiological parameters. Displayed are the line of unity (dotted), the coefficient of determination (R^2^) and the statistical significance (p).

**Fig. 7:**
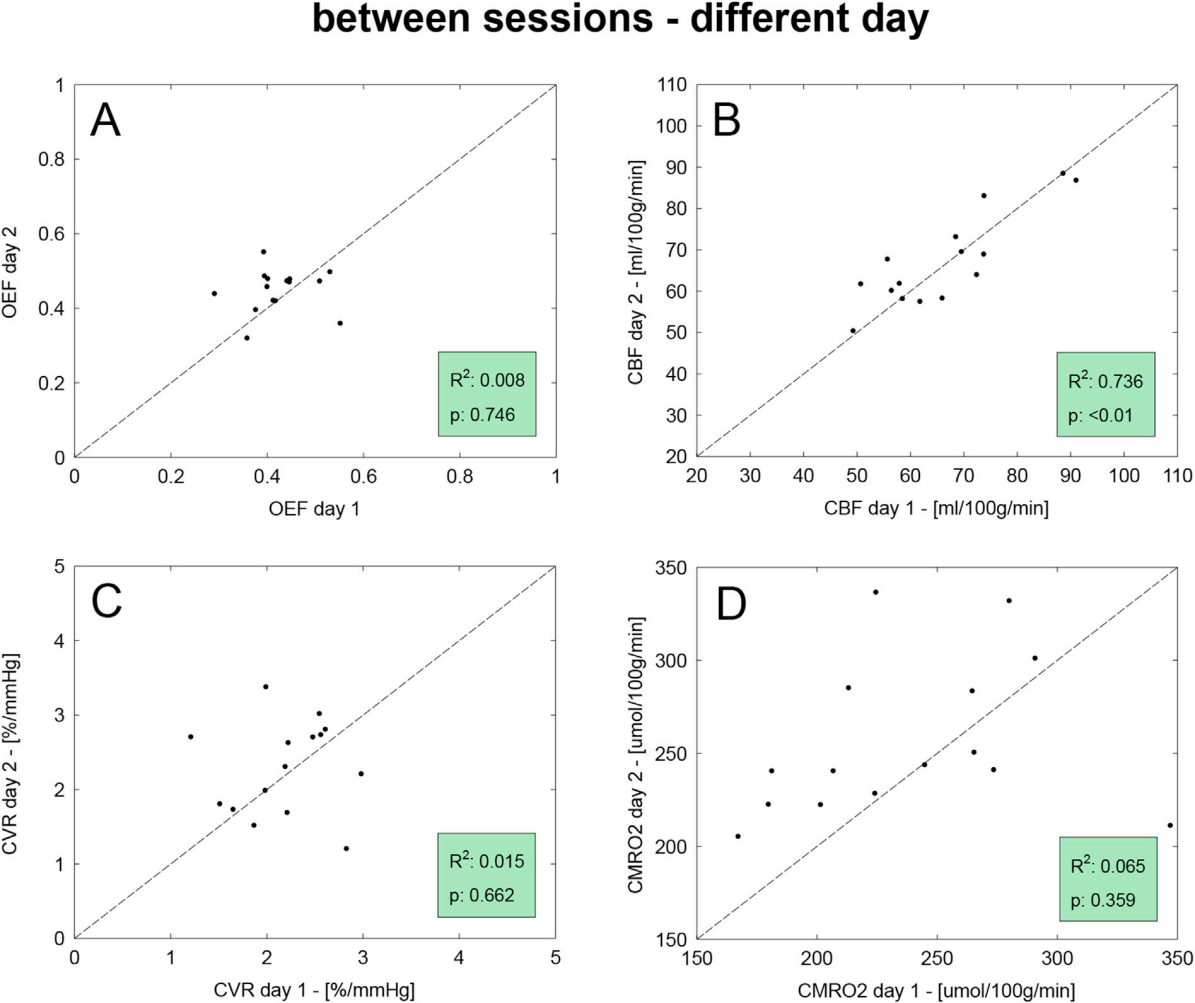
Results of the repeatability analysis on the between-sessions dataset with measurements acquired in different days. Scatterplots for the correlation analysis between the two sets of measurement (denoted as 1 and 2), for all four estimted physiological parameters. Displayed are the line of unity (dotted), the coefficient of determination (R^2^) and the statistical significance (p).

Results of the correlation analysis for the between sessions datasets are reported in Fig. 6, Fig. 7 for same and different days respectively. Values of correlation are overall lower than for the within-session dataset, with results from the same day found to be higher than those from different days. In both these last two cases, the correlation is particularly good for CBF with high values of the coefficient of determination (R^2^ > 0.7). Satisfactory levels of agreement are also reported for CVR in the case of between, same-day, while for the other estimated parameters the agreement is poorer.

CV indices reported in Fig. 4 D,F appear similar among the two instances of the between-sessions repeatability. Values of CV_inter_ are generally higher, ranging between about 25% for CVR to about 15% for OEF. CV_intra_ shows values (mean ± std.) of 10.8 ± 10% for OEF, 4.4 ± 2.7% for CBF, 12.5 ± 9.1% for CVR and 11.2 ± 9.7% for CMRO_2_ in the between sessions - same day, while of 10.5 ± 9.7% for OEF, 5.5 ± 4.7% for CBF, 17.5 ± 17.9% for CVR and 12.3 ± 10% for CMRO_2_ in the between sessions - different day. The distribution of CV_intra_ indices generally shows lower performances and outliers up to about 55% in the case of different days.

ICC_spatial_ indices are found to be overall consistent among same day and different day acquisitions for all four parameters, with mean values typically above 0.65 and a few outlying low values for the latter case (Fig. 4 C,E). Results are more varied for the bulk analysis, with values of ICC_global_ remarkably high for CBF in both cases, mostly “good” for CVR for the same day case, while “poor” for the rest, with the different day instance reporting the worse performances. In general, the performances reported are lower than for the within-session analysis.

The correlation analysis between the similarity of the respiratory traces in the two sessions and the estimated indices of CV_intra_ for the different physiological parameters did not show any significant results for the between sessions datasets (results not shown).

#### Voxel-wise results

**Fig. 8:**
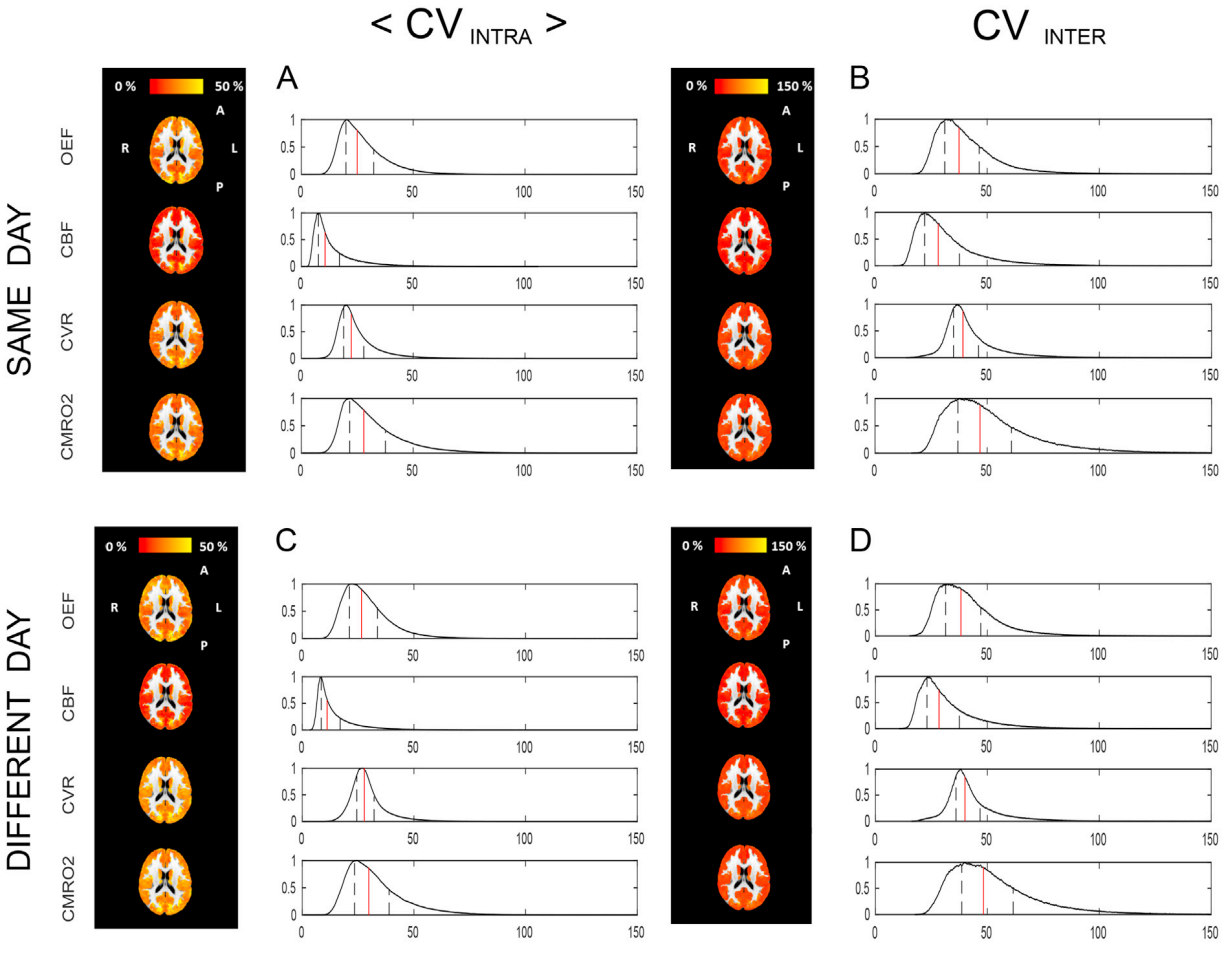
Voxel-wise CV indices calculated from the between-session dataset for the same day (A,B) or different day (C,D) case. Axial view of the calculated maps for each physiological parameter and relative histograms showing the distributions of the calculated values (in red the median and delimited in black the interquartile range). <CV_intra_> is the mean across subjects of the intra-subject CV and CV_inter_ is the inter-subjects CV.

Maps of the CV indices and normalised histograms for both same day- and different day-, between sessions repeatability are reported for each parameter in Fig. 8. Notably, for all physiological parameters areas of interface between grey matter and different structures and areas of the occipital lobe present higher CV values. As for the within session case, CBF shows the lowest variability, with values mostly homogeneous across parameters apart from a few areas, while CVR shows high <CV_intra_> and CV_inter_ indices with less regular distributions in space. Histograms show that for all parameters distributions of <CV_intra_> (Fig. 8 A,C) are shifted towards lower values compared to CV_inter_ (Fig. 8 B,D). The distributions are instead very similar when comparing indices obtained in acquisitions from the same day (Fig. 8 A,B) against acquisitions in different days (Fig. 8 C,D), with only a slight increase in the latter case.

Finally Figure S4 in the Supplementary Material section shows maps of the ICC index for each parameter and normalised histograms. In both cases the spatial distribution of the ICC results is in agreement that found for CV indices, with maps from the same day (Figure S4, A) very similar to maps from different days (Figure S4, B) and CBF results showing the higher performance in general. This is supported by the relative histograms, with only CVR values resulting appreciably higher for same-day acquisitions, while in contrast CBF values being even higher for different-day acquisitions. In general ICC values appear lower than the within-session case, with most distributions having median values below 0.5 and small but non-negligible proportion of negative values.

## Discussion

The present study aims to quantify the repeatability of a novel estimation approach based on a dcFMRI experiment, providing reference data characterising variability of CBF, CVR, OEF and CMRO_2_ estimates and thus informing the design of future dcFMRI studies. An analogous study recently evaluated the bulk and regional reproducibility of physiological parameters including OEF and CMRO_2_ estimates obtained with a similar dcFMRI calibration approach (Lajoie et al., 2016). In the present study we provide additional assessments of repeatability by comparing within and between (same- and different-day) session variability using two groups of subjects. Moreover, maps of the spatial distribution of the variability were obtained from a voxel-wise analysis.

With regards to the within-session dataset, grey matter values estimated with our forward model show an overall consistency of the results between the sets of measurements taken at two time points. Measured global grey matter values of 0.38 ± 0.08 (mean ± std) for OEF are in agreement with our previous reports (0.42 ± 0.12 (Wise et al., 2013),) or from other centres, with typical values for other MR methods ranging between 0.26 (Bolar and Rosen, 2011) and 0.435 (Fan et al., 2016). Notably, the variability of the reported whole brain estimates of OEF performs well compared to results from recent similar studies with dual calibrated fMRI approaches. Values of 0.43 ± 0.08 and 0.39 ± 0.06 in young (N = 28) and older (N = 45) subjects were found by De Vis et al. (De Vis et al., 2015), values of 0.435 ± 0.14 and 0.423 ± 0.17 were reported by Fan and colleagues for different application of the QUO2 method (N = 11 (Fan et al., 2016),) and finally values of 0.37 ± 0.06 were shown by Lajoie and colleagues (N = 8 (Lajoie et al., 2016),) again with QUO2.

Global grey matter measures of CBF (56 ml/100 mg/min) tend to be higher than what typically reported for MRI and PET studies (41 ml/100 mg/min (Bulte et al., 2012), 42 ml/100 g/min (Ibaraki et al., 2010)), but they are consistent with those from our previous study and similar ones, especially when considering young cohorts (56 ml/100 mg/min (Wise et al., 2013), 52 ml/100 mg/min (Gauthier and Hoge, 2012) and 63 ml/100 g/min (Ances et al., 2009)). Estimates of 2.6 ± 0.47%/mmHg for CVR lie on the lower side of the typical range of values obtained for comparable CO_2_ challenges in most of the MRI literature (between 5.15 ± 1.1%/mmHg (Bulte et al., 2012) and 2.82 ± 1.21%/mmHg (Heijtel et al., 2014)).

Finally mean CMRO_2_ values of 183 ± 49 μmol/100 g/min are comparable to reported values obtained with the dual calibrated fMRI method previously presented by our lab (184 ± 45 μmol/100 g/min (Wise et al., 2013)), other calibrated fMRI methods (145 ± 30 μmol/100 g/min (Gauthier and Hoge, 2012) and 155 ± 39 μmol/100 g/min (Bulte et al., 2012)) and values of 182 ± 12 μmol/100 g/min (Liu et al., 2013), 158 ± 18 μmol/100 g/min (Fan et al., 2012) and 157.4 ± 19.7 μmol/100 g/min (Roland et al., 1987) obtained with different MR methods and PET. In terms of variability around the mean value, our results are comparable with estimates of 181 ± 60 and 133 ± 43 μmol/100 g/min in young (N = 28) and older (N = 45) subjects respectively recently reported by De Vis and colleagues (De Vis et al., 2015) and with the values of 143 ± 34 shown by Lajoie and colleagues (Lajoie et al., 2016). Nevertheless in our study a few subjects show substantial changes between the two time points or outlying values, highlighting a degree of noise in the estimates, especially with regards to CVR and CMRO_2_.

The correlation analysis for the within session dataset shows a generally good level of correlation between the estimates, although highlights less than optimal performances in the cases of CVR and CMRO_2_. In particular, in Fig. 3-C,D few subjects appear as outliers. The main cause can be found considering the nature of the measurements, as both are derived from other estimates: CVR as the ratio between percent change in CBF and absolute changes in PetCO_2_ while CMRO_2_ as the product of OEF and CBF. This means that they are particularly sensitive to cumulative effects of noise in the original measurements.

ICC indices provide further quantification of the absolute agreement between the estimates: high values for the ICC_global_ index support the good performance found in the correlation analysis, while calculated ICC_spatial_ indices inform about consistency at a voxel-wise level. As expected, ICC_spatial_ is generally lower than ICC_global_ because averaging the estimate across grey matter allows some of the noise contributions to be reduced. In fact it might be argued that the good agreement of the estimates at a grey matter level is simply due to the averaging operated on a possibly wide range of noisy and non-informative estimates. Our analysis gives evidence that this is not the case: in fact ICC_spatial_ indices and the maps of ICC show that estimates are generally spatially consistent also at a voxel-wise level.

A further understanding of the variability in the within session data is given by the calculated coefficients of variance. CV_inter_ and CV_intra_ indices measure the proportion of the variability in the estimates originating from inter-subjects differences (such as normal distribution of physiological parameters in the cohort) and intra-subject differences (more related to measurement error, on the assumption of stable physiology). As values of CV_intra_ are found to be generally lower than CV_inter_, this means that the method applied is accurate enough to capture the single subject's physiology. Moreover, grey matter CV_intra_ values of 6.7 ± 6.6% (mean ± std.) for OEF, 6.9 ± 5.9% for CBF and 12 ± 9.6% for CMRO_2_ are comparable with those reported in PET literature for other methods aiming at estimating brain metabolism and haemodynamics across brain (5.7 ± 4.4%, 8.4 ± 7.6% and 5.3 ± 3.9% respectively for (Coles et al., 2006) and 9.3%, 8.8% and 5.3% respectively for (Bremmer et al., 2011). With regards to MRI, results are higher than those reported for bulk estimates coupling TRUST measurements and phase contrast imaging for CBF (3.2 ± 1.2%, 2.8 ± 0.8% and 3.8 ± 1.4% respectively (Liu et al., 2013)) but comparable with the values reported for the only fMRI calibrated study that addressed the issue of repeatability so far (about 4%, 13.5% and 15% respectively with QUO2 (Lajoie et al., 2016)). It is worth noting that in both this last example and our study, bulk grey matter results are obtained excluding voxels in which the estimation algorithm fails to converge to a solution or presents invalid estimates. The proportion of included voxels of the total considered is 88.5 ± 6% (m ± std) for that study (see Table 3 in (Lajoie et al., 2016)) and it is 96 ± 2% for our study. This is consistent with our previous report showing lower proportions of valid estimates obtained with the QUO2 analysis method compared to our forward modelling approach (Germuska et al., 2016). By neutralising the variability contribution from these problematic voxels, the repeatability estimates from Lajoie et al. (2016) may show higher performance but would have a decreased spatial coverage compared to those reported in our study.

A significant source of nuisance for the within-session repeatability appears to be related to the variations in the end tidal responses to the respiratory task between different acquisitions, at least for estimates of OEF. In fact the negative correlation found in Figure S1 in the Supplementary material indicates that changes in such response are correlated to higher CV_intra_ indices. Although, it is not possible to infer whether this is a causal relationship or such correlation is determined, for example, by a third underlying physiological variable.

The Bland-Altman plots (Supplementary material, Figure S2) visualize the relationship between the inter-subject and intra-subject variability, or measurement precision. Results confirm what was seen for the ICC and CV indices, that is a generally good agreement of the estimates in the two time points with a few outliers lowering the performance. It is also highlighted how values of OEF are mostly clustered around a physiological “average” value, whereas more varied values are found for CBF and CMRO_2_.

Voxel-wise CV indices are higher than those reported for bulk estimates, typically by a factor of 2 and 3 for the intra- and inter-subjects case respectively. Similarly, voxel-wise ICC indices show lower performance. This is expected due to the higher spatial resolution but it tells us about the spatial distribution of the variability in the estimates. In particular they show that the low CV indices calculated at a bulk level for OEF and CBF are representative of the voxel-wise distribution of these indices. Maps of CMRO_2_ further support the notion that the precision of the estimates is degraded by the combination of both OEF and CBF variability.

Results from the between-sessions analysis give us a further insight into the repeatability of the measurements obtained with our method for applications with acquisitions obtained during the same day but in different scanning sessions or in two different days. Compared to the within-session case, levels of repeatability are expected to decrease due to increasing levels of experimental variability, related to running two different sessions in the MRI scanner, and enhanced intra-subjects physiological variability (in the between, different day case), with possible variations in the participants' haemodynamic and metabolic state across time. In fact performance does decrease somewhat, with the lowest repeatability being between day for CMRO_2_, whose estimates subject to the cumulative effects of errors in CBF and OEF measurements. Notably, the correlation of OEF estimates shown in Fig. 6, Fig. 7 appears to be particularly poor. This could be driven by the relatively low physiological variance in true values of OEF rather than by the accuracy of our methods. In fact while values of CBF are found to vary considerably in the healthy brain depending of multiple factors such as age and gender, OEF typically varies within a narrower range of values, with an average of about 0.4 (in the healthy brain (Buxton, 2009), this being also observed for the within-session dataset and visible in Figure S2,A). This would manifest as scatterplots with isotropic distributions (rather than aligned along the unitary line) despite relatively low levels of CV indices, as reported by our analysis.

A first caveat in the present study relates to the application of our method under the assumption of isometabolism during hypercapnia and hyperoxia when performing respiratory tasks. Studies on the dependence of CMRO_2_ on altered arterial CO_2_ and O_2_ levels have found variable results, with some of the more relevant ones pointing at a decrease in metabolism with both hyperoxia and hypercapnia (Xu et al., 2012, Xu et al., 2011). An eventual deviation from isometabolism during these conditions would translate into bias on the estimates from calibrated fMRI models, as reported by studies from our and other groups (Merola et al., 2016, Blockley et al., 2015). In particular, we would expect values of OEF to be overestimated if CO_2_ lowered O_2_ metabolism, while values of OEF would be underestimated if O_2_ lowered O_2_ metabolism (Merola et al., 2016). Due to the form of the forward model, estimates of CBF are not expected to be affected by the violation of isometabolic conditions. Although still the subject of discussion in the field, this is a commonly adopted assumption for calibrated fMRI methods.

Another limitation arises from the precision of the estimates obtained with the forward model. As previously discussed, grey matter values reported are generally consistent with those found in literature. Repeatability of the measurement, quantified with correlation analysis and ICC calculations, has been shown to be overall satisfactory for the within-session repeatability, both at a bulk and voxel-wise level, the worst performances being related to the inherently noisiest derived parameters, i.e. CVR and CMRO_2_. CV indices are instead higher than those reported in literature for alternative MRI bulk measurements, as previously discussed. Although this does not represent a major limitation as in most cases values of CV_intra_ are lower than CV_inter_, indicating that the estimation precision of a subject's parameters is still good enough not to be confounded by the cohort's variability. In fact we should note that the estimates of four physiological parameters presented here have a voxel-wise resolution. Therefore, a trade-off between repeatability and spatial resolution has to be considered when comparing them to other methods only allowing bulk estimates of fewer parameters such as TRUST (Lu and Ge, 2008) or susceptibility methods applied to major veins (Fan et al., 2012, Jain et al., 2010). In our approach while the reproducibility is dependent on many factors, it is heavily conditioned by the ASL signal, which shows the lower SNR compared to the BOLD-weighted measurements. Therefore possible strategies to improve reproducibility would be preferentially focused on enhancing the quality of the ASL signal, for example adopting a pCASL tagging scheme or background suppression.

The final issue originates from the Bayesian approach adopted for the estimates. In fact the use of priors could potentially bias the estimates towards pre-determined values (the priors themselves) rather than the real ones. This would translate into good repeatability and decreased variability in the data, but ultimately resulting in a loss in sensitivity to differences in individual physiology. This argument, however, seems to be contradicted by the evidence of physiologically meaningful variation of estimates across grey matter and the presence of outlying values. Furthermore, also findings from previous studies in our centre point against this possibility. In fact, increased sensitivity to physiological changes was shown for the forward model used compared to other approaches (Germuska et al., 2016) and significant changes in physiology were found after caffeine consumption despite fixed priors (Merola et al., 2017).

Finally, this work helps us to design future studies based on the same estimation framework. By way of example, we consider three study designs: i) within subjects, within-session (based on scan 1 and 2 from the within session dataset); ii) within subjects, between sessions (based on the baseline scans from the between session dataset); and iii) between subjects (based on scan 1 from the within session dataset and the first baseline scan from the between session dataset).

Considering the bulk grey matter values reported, a significance level of 5% and a statistical power of 80%, the sample sizes (N) needed to detect effect sizes of 15%, 20% and 25% in the three study designs described are shown in Table 1.

**Table 1:** Sample size needed to detect effect sizes of 15%, 20% and 25% in OEF, CBF and CMRO_2_ for three different study designs (significance level = 5%, statistical power = 80%).

	OEF			CBF			CMRO_2_		
	15%	20%	25%	15%	20%	25%	15%	20%	25%
(i) within subjects, within session	14	8	5	15	9	6	24	14	9
(ii) within subjects, between sessions	8	4	3	11	6	4	12	7	5
(iii) between subjects	20	11	7	28	16	10	40	23	15

The lower numbers for case ii) compared to case i) seem counterintuitive and may just arise from uncertainty in variance estimates from our samples. Nevertheless, these calculations, supported by the repeatability analysis previously presented, suggest that our approach can be usefully applied with practical sample sizes. In order to avoid large cohorts, experimental designs characterized by reduced variability in the data should be chosen, such as crossover and longitudinal.

In conclusion with this study we have characterised the variability of the estimates obtained with our dcFMRI method, showing an overall consistency with literature reports and a good level of repeatability. Performance varies for the different physiological parameters and according to spatial resolution and study design. In particular the information supplied by grey matter maps is of extreme interest for studies focused on the spatial distribution of brain physiology, despite some reliability limitations compared to methods supplying bulk measurements. The level of variability in the data suggest that the dcFMRI approach can be applied usefully for appropriate experimental designs with sample sizes typically found in MRI studies.

## Figures and Tables

**Table 2:** List of main physiological parameters involved in the modelling. For each of them the role in the estimation process (“measured”, “estimated”, “calculated” or assumed “fixed”) and relative value (prior: [mean, standard deviation], fixed value or defining equation) and units are reported.

Parameter	Role in modelling	Value	Units
OEF	estimated	prior: [0.35,0.1]	–
CBF	estimated	prior: [60,Inf]	ml/100 g/min
CVR	estimated	prior: [3, 0.774]	%/mmHg
K	estimated	prior: [0.07, 0.087]	–
CMRO_2_	calculated	(Eq. A-11)	μmol/100 g/min
PaCO_2_	measured	–	mmHg
PaO_2_	measured	–	mmHg
M_0,blood_	measured	–	–
θ	fixed	0.06	–
φ	fixed	1.34	ml/g
Hb	fixed	15	g/dl
T_1b_	calculated	(Eq. A-9)	s
b; c (Eq. A-9)	fixed	-5x10^−4^; 1.78	s/mmHg; s
t_max_(Eq. A-10)	fixed	0.2	s

## Appendix

The estimation framework used in this work for both the within-session and the between-sessions datasets is described with more detail in the original paper from our centre (Germuska et al., 2016) and has already been applied in its Bayesian variant in a recent publication (Merola et al., 2017). The code used for the estimates is available in the Cardiff University data Catalogue at https://doi.org/10.17035/d.2015.100126.

A forward signal model is constructed by combining a detailed description of the arterial spin labelling (ASL) signal developed by Woolrich and colleagues (Woolrich et al., 2006) and a model of the BOLD signal developed in our centre (Wise et al., 2013). Here we summarize the equations relating the estimated parameters OEF, CBF, CVR and CMRO_2_.

The total MR signal S_TOT_ resulting from a dual-gradient echo (GRE) imaging sequence with a PASL PICORE, QUIPSS II scheme for ASL signal can be expressed as:

(A-1) $$S_{TOT}=\;S_{ASL}\;e^{-TE\;R_{2,0}^{*}\left(1+\Delta R_{2}^{*}/\;R_{2,0}^{*}\right)}$$

colleagues (Wise et al., 2013 Where SASL is the ASL signal and the BOLD contribution is accounted for by changes in transverse relaxation rate R2*. With regards to the latter, it can be expressed following the model first proposed by Wise and colleagues (Wise et al., 2013) and then optimised (Merola et al., 2016) as:

(A-2) $$\Delta R_{2}^{*}=\;K\;{\left[dHb\right]}_{0}\;\left[f^{\theta }\left(\frac{\left[dHb\right]}{{\left[dHb\right]}_{0}}\right)-1\;\right]$$

and

(A-3) $$f=\;\left(1+\frac{CVR\;\;\Delta PaCO_{2}}{100}\right)$$

Where [dHb] is the deoxy-haemoglobin concentration (_0_ for baseline), PaCO_2_ is the arterial partial pressure of O_2_, CVR is the cerebrovascular reactivity (in %ΔCBF/mmHg_CO2_), θ is an optimised fitting parameter and K is a lumped parameter depending on cerebral blood volume, field magnitude and geometry. Then OEF can be calculated from the expression for [dHb]/[dHb]_0_ proposed by Wise and colleagues (Wise et al., 2013):

(A-4) $$\frac{\left[dHb\right]}{{\left[dHb\right]}_{0}}=\;\frac{1}{f}-\frac{1}{{\left[dHb\right]}_{0}}\left[\frac{1}{\phi }\left(CaO_{2}-\frac{1}{f}CaO_{2,0}\right)\;+\;\left[Hb\right]\left(\frac{1}{f}-1\right)\right]\;$$

Where [Hb] is the haemoglobin concentration, φ is the O_2_ carrying capacity of haemoglobin (1.34 ml O_2_/g_Hb_, see Table 2) and CaCO_2_ is the arterial O_2_ content. OEF is then related to [dHb]_0_ by:

(A-5) $${\left[dHb\right]}_{0}=\left[Hb\right]\cdot \;OEF$$

The ASL contribution S_ASL_ can also be expressed, following Woolrich model (Woolrich et al., 2006), as a sum of a static component (S_s_) and a component due to perfusion (S_b_):

(A-6) $$S_{ASL}=\;S_{s}+\;S_{b}$$

where the static component is expressed in terms of changes in voxel magnetization M:

(A-7) $$S_{s}=\;M_{0}\left(1+\Delta M/M_{0}\right)$$

So that changes in M_0_ are assumed to derive from a change in blood volume and water exchange. The perfusion component is then expressed in terms of changes of CBF and the kinetic PASL model:

(A-8) $$S_{b}=\;M_{0,blood}\;CBF\;\left(1+\frac{CVR\;\cdot \;\Delta PaCO_{2}}{100}\right)+\left\{\left[TI_{1}\left(Rp_{n}+\left(1-R\right)\right)\right]+\left[\left(TI_{2}-TI_{1}-\delta t\right)\cdot \;\left(1-e^{-\left(TI_{1}-TI_{2}\right)/T_{1b}}\right)+\delta t\right]\right\}$$

Where M_0,blood_ and T_1b_ are respectively the baseline magnetization and the longitudinal relaxation time of blood, R =  $e^{-TI_{2}/T_{1b}}$ and with p_n_ = 1 for control and p = −1 for tag. Finally TI_1_, TI_2_ and δt are the times time to saturation, time to imaging and transit time defined by the QUIPSS II tagging scheme. The relationship between PaO_2_ and arterial T_1b_ is taken to be linear (as per Ma and colleagues (Ma et al., 2014),) and described by:

(A-9) $$T_{1,b}=b\;PaO_{2}+c$$

The unknown parameters were fixed to literature values, with b = -5x10^−4^ (extrapolated from Ma and colleagues (Ma et al., 2014),) and c = 1.78 (from Lu and colleagues, (Lu et al., 2004), see Table 2). With regards to the contribution of PetO_2_ and to PetCO_2_ to calculated CaO_2_ and ΔPaCO_2_ respectively, local variation of the haemodynamic response (*hr(t)*) was allowed modelling each of them separately as gamma-variate functions:

(A-10) $$hr\left(t\right)=\;t_{max}^{-\eta_{i}}\;e^{\eta_{i}}\;t^{\eta_{i}}\;e^{-\eta_{i}t/t_{max}}$$

with t_max_ (fixed to 0.2 s, see Table 2) denoting the time of peak and η_i_ free parameter determining the rise and fall times of the response (with i = CO_2_, O_2_). Finally CMRO_2_ is calculated as:

(A-11) $$CMRO_{2}=OEF\;CBF\;C_{a}O_{2}$$

Finally, Table 2 reports the main physiological parameters involved in the modelling with information about their role in the estimation process and values.

## Appendix A Supplementary data

FigS1 FigS1

FigS2 FigS2

FigS3 FigS3

FigS4 FigS4

## Footnotes

Appendix A Supplementary data related to this article can be found at https://doi.org/10.1016/j.neuroimage.2018.02.020.
