# Supplementary figures and images for: Assessing the repeatability of absolute CMRO2, OEF and haemodynamic measurements from calibrated fMRI
Source: Neuroimage. 2018 Jun;173:113–26. doi: 10.1016/j.neuroimage.2018.02.020 (PMC6503182; doi:10.1016/j.neuroimage.2018.02.020)

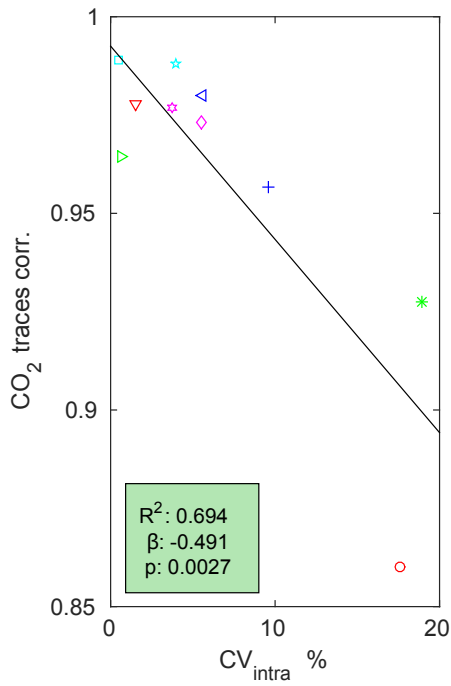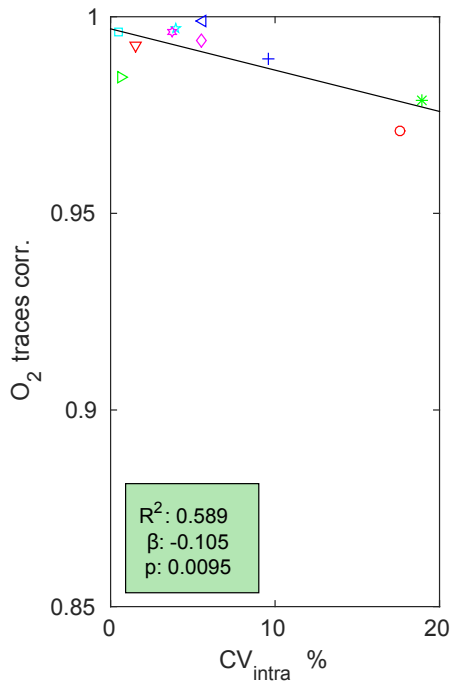

Supplement: FigS1 [file mmc1.pdf]

OEF

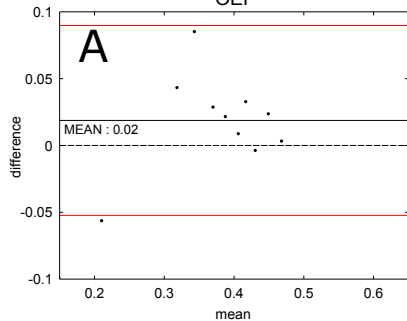

CBF

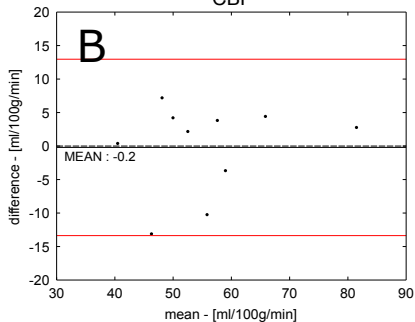

CVR

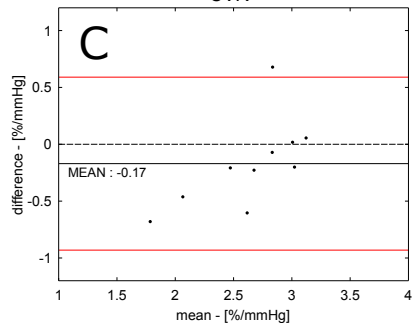CMRO<sub>2</sub>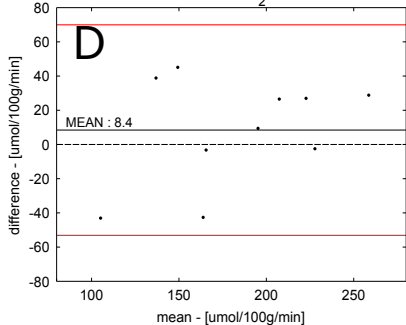

Supplement: FigS2 [file mmc2.pdf]

# ICC

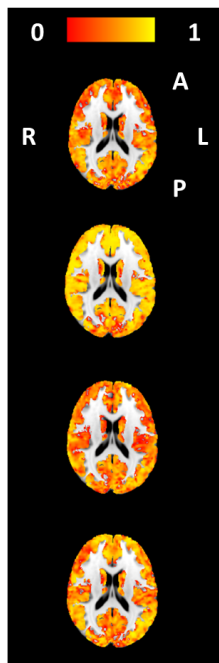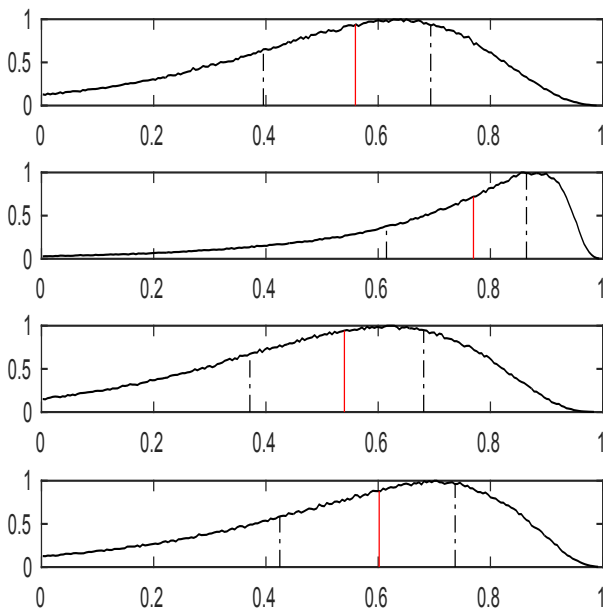

Supplement: FigS3 [file mmc3.pdf]
