# Supplementary material for: Assessing the repeatability of absolute CMRO2, OEF and haemodynamic measurements from calibrated fMRI
Source: Neuroimage. 2018 Jun;173:113–26. doi: 10.1016/j.neuroimage.2018.02.020 (PMC6503182; doi:10.1016/j.neuroimage.2018.02.020)
Supplement: FigS4 [file mmc4.pdf]

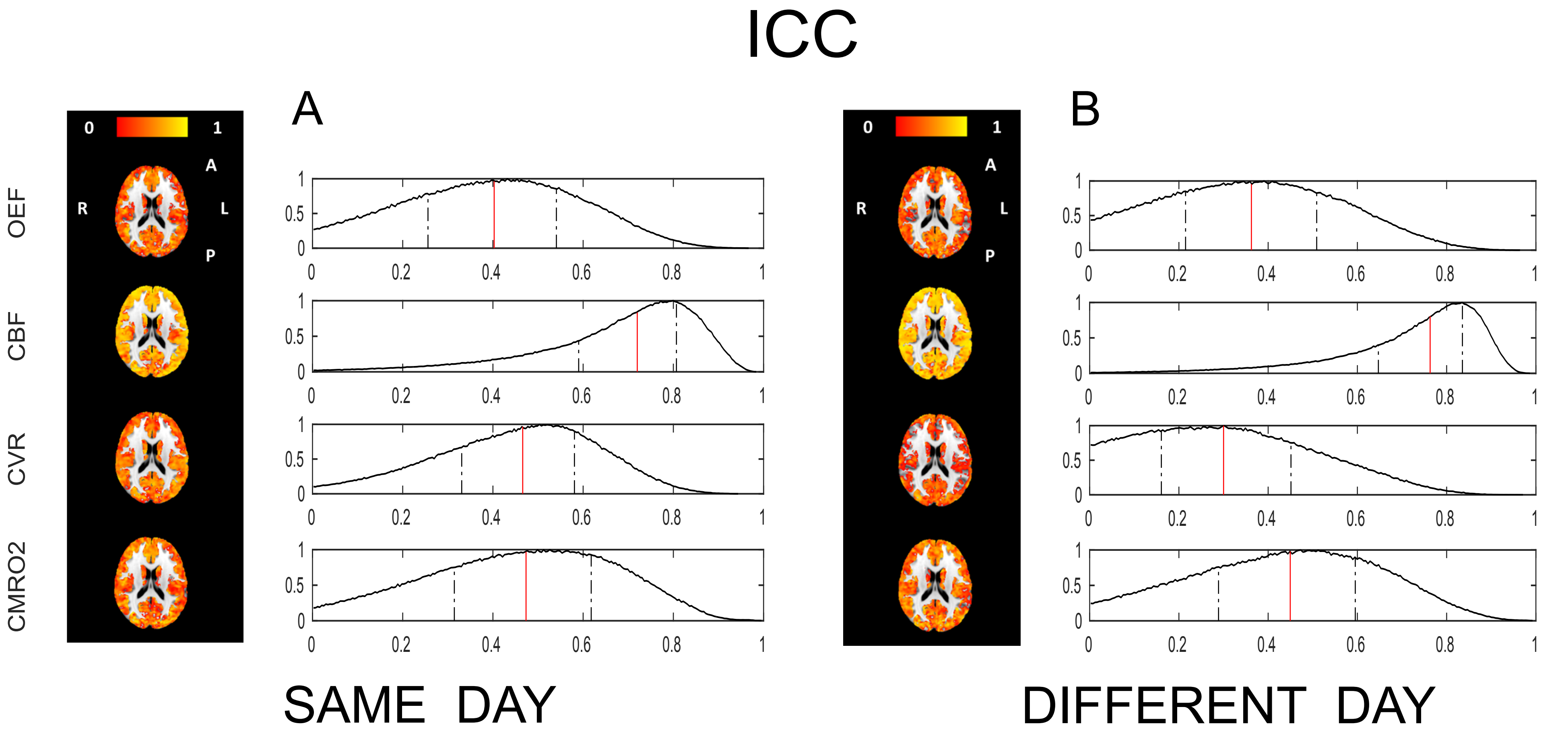

ICC
A
B
0
1
A
R
L
P
OEF
CBF
CVR
CMRO2
1
0.5
0
0.2
0.4
0.6
0.8
SAME DAY
DIFFERENT DAY
